# Community-based childhood obesity prevention intervention for parents improves health behaviors and food parenting practices among Hispanic, low-income parents

**DOI:** 10.1186/s40608-018-0188-2

**Published:** 2018-03-27

**Authors:** Laura Otterbach, Noereem Z. Mena, Geoffrey Greene, Colleen A. Redding, Annie De Groot, Alison Tovar

**Affiliations:** 1 Department of Nutrition and Food Sciences, University of Rhode Island, Fogarty Hall, 41 Lower College Rd, Kingston, RI 02881 USA; 2 Cancer Prevention Research Center and Department of Psychology, University of Rhode Island, Chafee Hall, 142 Flagg Road, Kingston, RI 02881 USA; 3 Institute for Immunology and Informatics, University of Rhode Island, Shepard Building, 80 Washington Street, Providence, RI 02903 USA

## Abstract

**Background:**

Given the current prevalence of childhood obesity among Hispanic populations, and the importance of parental feeding behaviors, we aimed to assess the impact of the evidence-based Healthy Children, Healthy Families (HCHF) intervention on responsive food parenting practices (FPPs) in a low-income Hispanic population.

**Methods:**

This community-based pilot study used a non-experimental pre/post within-subjects design. Parents (*n* = 94) of children aged 3–11 years old were recruited to participate in an 8-week, weekly group-based intervention. The intervention was delivered to nine groups of parents by trained paraprofessional educators over a two-year period. Children participated in a separate curriculum that covered topics similar to those covered in the parent intervention. Parents completed self-administered pre/post surveys, which included demographic questions, seven subscales from the Comprehensive Feeding Practices Questionnaire, and the 16-item HCHF Behavior Checklist. Descriptive statistics and paired samples t-tests were used to analyze data from parents that completed the intervention.

**Results:**

Fifty-two, primarily Hispanic (93%) parents completed the intervention (39% attrition rate). For parents who completed the intervention, there was a significant increase in one of the feeding practice subscales: encouragement of balance and variety (*p* = 0.01). There were significant improvements in several parent and child diet and activity outcomes (*p* ≤ 0.01).

**Conclusions:**

Although attrition rates were high, parents completing the study reported enjoying and being satisfied with the intervention. For parents who completed the intervention, reported ‘encouragement of balance and variety’, in addition to several health behaviors significantly improved. Larger studies utilizing an experimental design, should further explore the impact of the HCHF curriculum on improving certain FPPs and health behaviors that contribute to obesity.

## Background

Prevention of childhood obesity is an ongoing public health priority. From 2011 to 2014, 17% of children and adolescents in the United States (U.S.) were obese [1], with Hispanic children experiencing a greater prevalence of obesity compared to non-Hispanic White children (22% vs. 14%, respectively) [1]. To reduce these racial/ethnic disparities, obesity prevention programs and interventions for Hispanic parents are urgently needed [1–4]. In addition, more research is needed on community-based interventions that actively engage parents around their child's healthy eating, physical activity and the home environment [4, 5]. Given that parents influence their children’s health behaviors and environment early in life, involving them in childhood obesity prevention is critical [4, 6–11].

Parents influence their child’s health behaviors through the home environment and their parenting practices [4, 6–18]. Food parenting practices (FPPs) are strategies used by parents to influence both the amount and types of food a child eats [11, 13, 18, 19]. It is important to teach parents about responsive feeding practices, such as role modeling healthy eating behaviors for their child; involving their child in food decisions such as grocery shopping and meal preparation; and encouraging a balanced and varied diet with their child [11–13, 15, 17–24]. These responsive FPPs have been associated with healthier diets and body mass index (BMI), while non-responsive feeding practices such as restriction and pressure have been associated with lower quality diets and higher BMIs [11–13, 15, 17–24].

Although multiple interventions aim to prevent childhood obesity [14–16, 25], few have specifically targeted the use of responsive FPPs among high-risk populations [9, 10, 14, 16], such as low-income Hispanics. *Healthy Children, Healthy Families: Parents making a difference!* (HCHF) is an evidence-based curriculum designed to be delivered to parents of children 3–11 years of age that focuses on developing healthy lifestyle behaviors [26–28]. Goals of the HCHF curriculum include increasing parent knowledge and skills surrounding implementation of healthy family habits, which ultimately impact child health behaviors [26–29]. Previous studies utilizing the HCHF curriculum reported significant improvements in several parent and child health behaviors using the 16-item HCHF Behavior Checklist (HCHF-BC) [26, 28–30]. While the checklist was developed specifically for the HCHF intervention, it does not comprehensively measure changes in FPPs using validated tools [28, 30]. Therefore, the goal of this pilot study was to assess if parents from a primarily Hispanic and low-income community, who participated in a childhood obesity intervention (HCHF) improved their responsive FPPs, specifically, 1) modeling healthy eating behaviors to their child, 2) encouraging a balanced and varied diet to their child, 3) involving children in food decisions, and 4) teaching their children about nutrition. In addition, the study aimed to assess changes in parent and child behaviors related to dietary intake and activity, using the 16-item HCHF-BC.

## Methods

### Study design

The pilot study utilized a non-experimental, pre/post within-subjects design in a community-based setting. The study involved a community partnership with Clinica Esperanza/Hope Clinic (CEHC), a clinic providing free healthcare services and programs to uninsured adults. The intervention was delivered by formally trained community paraprofessionals called *Navegantes.* A total of five *Navegantes* delivered the HCHF intervention to participants over the 2-year study period, with 2-3 *navegantes* teaching or facilitating each lesson at a time. All *Navagantes* were women from the surrounding community that were employed at CEHC. Over the course of 2 years (2014-2016), parents of 3-11-year-old children were recruited to participate in the study, which was framed as a community program entitled ‘Niños Activos y Sanos: Healthy & Active Children’. All of the protocols in the study were approved by the University of Rhode Island Institutional Review Board.

### Participants and recruitment

Both parents and primary caregivers, such as grandparents, (all referred to as ‘parents’ throughout this manuscript) with a child between the ages of 3-11 years were recruited. The target population were parents living in Olneyville and South Providence, Rhode Island where the median household income is $17,538, and 61% of the population is Hispanic [31].

Both in-person recruitment at different community settings (i.e. local parks, churches, community centers, events, etc.) and recruitment fliers were distributed throughout the community to recruit participants on a rolling basis from 2014 to 2016. In addition, researchers and *Navegantes* collaborated with community partners including other healthcare clinics and health-related programs to recruit parents for the study. Parents and/or primary caregivers were screened in-person or via telephone to determine eligibility. Participants were eligible to participate if they were a parent or primary caregiver of a child between 3 and 11 years of age at the beginning of enrollment and spoke English or Spanish. During the first year, 44 parents enrolled, 50 in year two (*N* = 94), with a total of nine groups of parents completing the intervention over the 2 year period. Groups occurred sequentially over the two-year study period. During the second year, the intervention was reduced by 1 week in effort to improve study retention.

### Intervention

*Navegantes* participated in a formal 2-day training prior to delivering the HCHF curriculum, which was delivered primarily in Spanish. Based on previous evidence, the HCHF curriculum was designed to provide parents with strategies to help children adopt behaviors that promote a healthy weight [26, 27]. Through problem-solving strategies and role-playing, the HCHF intervention highlights *‘paths to success’* (nutrition and physical behaviors) and *‘keys to success’* (parental strategies to facilitate progress on the path to healthy behaviors in families, which highlight several responsive FPPs, and encourage parents to use these practices at home) [26, 27].

For example, paths to success include ‘eating more fruits and vegetables’, ‘eating fewer high-fat and high-sugar foods’, ‘playing actively’, and ‘limiting television and computer time’ [26, 27]. Examples of keys to success include setting a good example for their child (modeling), and offering healthy choices within limits (guiding, or encouraging a balanced and varied diet) [26, 27].

Parents attended 90-min, weekly sessions of HCHF, which were conducted on Wednesday evenings, usually beginning at 5:30 pm. Written informed consent was obtained from all participants. Parents completed written informed consent forms to participate in the study and informed assent/written permission forms for their child if they were under the age of 7 years. Modified forms were used to allow children over the age of seven to better understand the study and provide written informed assent to participate. After consent was obtained from all participants, parents and children completed anthropometric measurements and parents then completed a written survey. Researchers were present during completion of consent forms and surveys to answer questions, assist parents who could not read or write, and provide clarification as needed. All study materials were available in both English and Spanish. The intervention was designed for the parents, given their role in shaping their child’s environment and behaviors. During the sessions however, childcare and nutrition lessons were provided to children if parents chose to bring them. Parents then returned to CEHC weekly, for a total of eight sessions to complete the intervention. At the last session, the same procedures were repeated to collect post-intervention data (with the exception of consent forms). Parents were compensated with a $10 gift card after the first session, and a $40 gift card following the last session. Each session also included a weekly prize (such as pedometers, mixing bowls, and spatulas) for parents and their children in addition to raffle prizes (such as food prep equipment, small kitchen tools).

### Measures

#### Anthropometrics

Standing height and weight measurements of each parent-child dyad were taken using standardized procedures [32], taken in duplicate. Parent’s BMI was calculated based on their height and weight. Pre and post-intervention BMI percentiles were calculated for children using age- and sex-specific references [32, 33].

#### Survey

The self-administered survey consisted of 84 questions and parents answered questions as it pertained to their child that was consented to participate in the study. Parents with more than one child between ages 3-11 were instructed to base their responses on their youngest child within that age range. The decision to do this was driven by the literature on the importance of shaping health behaviors early in life given that these track into later childhood [4, 6, 7, 9, 10, 16, 21].

Parents were asked to report on the following socio-demographic characteristics: age, sex, ethnicity, race, education level, number/ages of children, marital status, if they were born in the U.S., number of years in the U.S., employment status, health insurance status, annual household income, child date of birth, and child gender.

Reported food parenting practices were assessed using seven subscales from the previously validated CFPQ [34], including modeling (4 items; α = 0.79), involvement (3 items; α = 0.89), encouraging balance and variety (4 items; α = 0.72), and teaching about nutrition (3 items; α = 0.42). Response options, ranged on a scale from disagree (1), disagree slightly (2), neutral (3), slightly agree (4), to agree (5) [34]. Subscale means were calculated for the seven subscales, with a higher score on each subscale indicating greater agreement with the corresponding practice.

To assess frequency of parent and child health behaviors, including dietary and physical activity/screen time behaviors (11 items), and home environment/parenting behaviors (5 items), parents completed the self-reported 16-item HCHF-BC [30]. Each item was assessed using a 5-point response scale from least to most frequent options in a range of frequencies appropriate to each reported behavior [30]. For example, for some of the questions response options ranged from (1) once in a while, (2) 1-2 days each week, (3) 3-4 days each week, (4) 5-6 days each week, to (5) every day or from (1) almost never, (2) 1-3 days each week, (3) 4-6 days each week, (4) once each day, to (5) 2 or more times each day. Mean scores for each item were calculated for analysis.

In addition to study objectives focused on parental feeding and diet and activity measures, a brief evaluation survey (14 questions) was provided to parents at the end of the final HCHF session, in effort to obtain their opinions and feedback on the program. Twenty participants that completed the study filled out an evaluation survey (surveys were provided in both English and Spanish).

### Statistical analysis

*Post-hoc* analysis was completed to assess if there were significant changes between demographic variables for study completers vs. non-completers. Chi-square tests for categorical variables and an ANOVA for continuous variables were completed to compare demographics between completers and non-completers. Paired samples t-tests were performed to assess for statistically significant changes pre/post intervention for seven CFPQ subscales and the 16-item HCHF-BC. Given that this was a pilot study to assess the preliminary efficacy of the intervention, it was not adequately powered for multivariate analyses. To account for multiple comparisons, a conservative significance level was set post hoc for the t-tests at *p* ≤ 0.01. The datasets used and/or analyzed during the current study are available from the corresponding author on reasonable request.

To assess parent participation, attendance was recorded at each session. Parents were considered study completers and were included in data analyses if they attended four or more of the eight sessions in year one, or three or more of the seven sessions in year two. To assess intervention fidelity (described as the extent to which the intervention is delivered as it was intended) [35], 59% of the HCHF sessions were observed by a trained research assistant using a previously-developed observation checklist. All statistical analysis was performed using SPSS version 23.

## Results

**Fig. 1:**
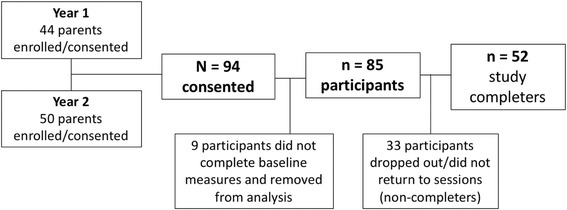
Flowchart for study recruitment & completion

Of the 94 parents who consented to participate over the 2 year study, nine did not complete baseline measurements, and were therefore excluded from analysis, leaving a total of 85 participants. Throughout the 2-year period, 33 parents dropped out of the intervention (i.e. did not return to the program sessions and/or did not complete post-intervention measures) and were therefore considered non-completers, leaving *n* = 52 participants with both baseline and post-intervention data (attrition rate of 39%). Figure 1 depicts a flow diagram showing the recruitment/consent process. Study completers were significantly older as compared to non-completers (39.9 vs. 34.4 mean years of age, respectively), (*p* = 0.031) (see Table 1).

**Table 1:** Baseline characteristics of NASA participants (*n* = 85)

Participant characteristics	All parents (*n* = 85)	Study completers (*n* = 52)	Non-completers (*n* = 33)
	*n* (%)	*n* (%)	*n* (%)
Sex			
Female	81 (95.3)	50 (96.2)	30 (90.9)
Age (mean±SD)	37.6±11.3	39.9±10.9^a^	34.4±11.1^a^
Hispanic/Latino			
Yes	78 (91.8)	49 (94.2)	29 (87.9)
Race (check all that apply)			
White	36 (42.4)	23 (44.2)	13 (39.4)
African-American	10 (11.8)	7 (13.5)	3 (9.1)
American Indian/Alaskan Native	1 (1.2)	0 (0.0)	1 (3.0)
More than once race	9 (10.6)	5 (9.6)	4 (12.1)
Wish not to answer/don’t know	10 (11.8)	4 (7.7)	6 (18.2)
*Did not answer/Missing*	19 (22.4)	13 (25)	6 (18.2)
Education			
Less than high school	25 (29.4)	13 (24.9)	12 (36.4)
High school graduate/GED	24 (28.2)	17 (32.7)	7 (21.2)
Post High School Trade/Technical school	9 (10.6)	7 (13.5)	2 (6.1)
Some college or higher	27 (31.7)	15 (28.9)	12 (36.4)
Living with Spouse			
No	45 (52.9)	26 (50.0)	19 (57.6)
Marital Status			
Never Married	26 (30.6)	12 (23.5)	14 (42.4)
Married	32 (37.6)	21 (41.2)	11 (33.3)
Divorced/Separated or Widowed	26 (30.6)	18 (35.3)	8 (24.3)
Born in the U.S.			
No	67 (78.8)	43 (82.7)	24 (72.7)
Years in the U.S. (mean±SD)	13.0±10.3	12.4±9.7	14.1±11.4
Employment Status			
Employed Full time (> 35 hrs/wk)	24 (28.2)	11 (21.2)	13 (39.4)
Employed Part time (< 35 hrs/wk)/Seasonally	21 (24.7)	14 (26.9)	7 (21.2)
Unemployed/Looking for work	25 (29.4)	17 (32.7)	8 (24.2)
Homemaker	13 (15.3)	10 (19.2)	3 (9.1)
Student	1 (1.2)	0 (0.0)	1 (3.0)
Health Insurance			
Yes	53 (62.4)	32 (69.6)	21 (63.6)
Annual Household Income			
$15,000 or less	43 (50.6)	29 (55.8)	14 (42.4)
$15,000 - $30,000	15 (17.6)	9 (17.3)	6 (24)
$30,000 - $45,000	8 (9.4)	3 (5.8)	5 (15.2)
More than $45,000	2 (2.4)	2 (3.8)	0 (0.0)
Parent Baseline BMI score (kg/m^2^)			
Underweight (< 18.5)	0 (0.0)	0 (0.0)	0 (0.0)
Normal Weight (18.5 – 24.9)	16 (19.2)	12 (22.8)	4 (12.0)
Overweight (25.0 – 29.9)	25 (30)	16 (30.4)	9 (27.0)
Obese (30.0 or higher)	38 (49.4)	23 (43.7)	17 (51.0)
Child Baseline BMI Percentile			
Underweight (<5th)	1(1.2)	1 (2.3)	1 (3.8)
Normal Weight (5th – <85th)	32 (37.7)	20 (46.5)	12 (46.2)
Overweight (85th - <95th)	12 (14.1)	9 (20.9)	4 (15.4)
Obese (≥95th)	40 (47.0)	13 (30.2)	9 (34.6)
Child Age (mean±SD)	5.9±2.8	5.8±2.5	5.9±3.3
Child Gender			
Female	46 (54.1)	28 (53.8)	18 (54.5)

Values above that do not add to 100% reflect missing data

Abbreviations: *NASA* Ninos Activos y Sanos/Healthy & Active Children (Name of the program), *SD* Standard deviation, *GED* General Education Diploma, *U.S.* United States, *Hrs* Hours, *Wk* Week, *BMI* Body Mass Index

^a^ Differences between variables for completers and non-completers were significant (*p* < 0.05), *p* = 0.031

Of the 85 parents at baseline, 94% were female and Hispanic with a mean age of 37.6 years. Less than a third (30%) of parents had less than a high school degree, over half (64%) reported an annual household income of $15,000 or less and the majority (79%) were not born in the U.S. (Table 1). At baseline, over 75% of the parents were either overweight or obese. Of the participating children, mean age was 5.9±2.8 years, 56% were female, and almost half (49%) were either overweight or obese. Intervention fidelity was high (97%), indicating that the *Navegantes* delivered the intervention as it was intended based on the HCHF curriculum protocol.

For responsive FPPs, there was a significant increase in the frequency of reported use of encouraging balance and variety (4.5 pre, 4.63 post, *p* = 0.01; Effect size (Cohens d = 0.263). There were increases in the frequency of other responsive FPPs including modeling, involvement, and teaching about nutrition, however these changes were not statistically significant. For outcomes related to non-responsive FPPs, changes were not significant (Table 2).

**Table 2:** Parent pre/post intervention FPPs using Subscales from the CFPQ (*n* = 52)

CFPQ subscale	Pre		Post		95% Confidence Interval of the Difference		Effect size (Cohens d)	*p*-value
	Mean	SD	Mean	SD	Lower	Upper		
Modeling	4.47	0.75	4.62	0.65	−3.59	0.07	0.20	0.17
Encouragement of Balance and Variety	4.50	0.52	4.63	0.56	−0.29	0.04	0.26	0.01*
Involvement	3.90	0.97	4.03	0.99	−0.49	0.24	0.12	0.48
Teaching About Nutrition	3.73	0.72	3.87	0.58	−0.34	0.06	0.21	0.17
Restriction for Health	3.80	0.88	3.86	1.04	−0.35	0.23	0.07	0.66
Restriction for Weight Control	3.04	1.05	3.17	1.07	−0.42	0.14	0.16	0.33
Food as Reward	3.09	0.96	2.93	0.95	−0.19	0.50	0.14	0.38

* *p* ≤ 0.01 denoted statistically significant difference between pre/post measures from the CFPQ subscale

For changes in parent and child behaviors related to dietary intake and physical activity, there were significant increases in frequency reported intake of fruit (2.9 pre, 3.7 post; *p* < 0.001), vegetable (2.9 pre, 3.6 post; *p* < 0.001) and low-fat dairy products for parents, (3.1 pre, 3.7 post; *p* = 0.003). Parents significantly increased the frequency of their own reported physical activity (2.5 pre, 2.9 post; *p* = 0.006) (Table 3).

**Table 3:** Parent-reported pre/post intervention diet and physical activity outcomes from the HCHF-BC (*n* = 52)

Item on HCHF-BC	Pre		Post		95% Confidence Interval of the Difference		*p*-value
	Mean	SD	Mean	SD	Lower	Upper	
Parent Fruit Intake	2.98	1.48	3.74	1.17	−1.14	−0.38	0.000**
Parent Vegetable Intake	2.92	1.34	3.58	1.05	−0.97	− 0.35	0.000**
Parent Soda Intake	1.72	1.17	1.45	0.90	−0.03	0.59	0.079
Parent Low-Fat Dairy Intake	3.12	1.36	3.66	1.36	−0.88	−0.20	0.003*
Parent Physical Activity	2.45	1.51	2.92	1.46	−0.80	−0.14	0.006*
Child Fruit Intake	4.04	1.03	4.10	1.13	−0.39	0.27	0.714
Child Vegetable Intake	2.88	1.38	3.18	1.20	−0.66	0.05	0.087
Child Low-Fat Dairy Intake	3.28	1.34	3.72	1.20	−0.84	−0.04	0.033
Child Soda Intake	1.55	0.89	1.55	1.10	−0.27	0.27	1.00
Child Physical Activity	2.90	1.49	3.53	1.30	−1.04	−0.22	0.003*
Child Screen Time	2.20	0.78	2.02	0.71	−0.05	0.41	0.118

*p ≤ 0.01, **p ≤ 0.001 denoted statistically significant difference between pre/post measures from the HCHF Behavior Checklist

There were also changes in measures related to the home food environment. Reported fruit availability significantly increased (4.3 pre, 4.6 post; *p* = 0.009), while energy dense snack availability (2.4 pre, 1.8 post; *p* = 0.001) and fast food intake (1.7 pre, 1.3 post; *p* = 0.003) significantly decreased. The changes in reported parental use of food autonomy (parent letting their child decide how much to eat), and the frequency of family meals were not significant (Table 4).

**Table 4:** Parent-reported pre/post intervention parenting and home food environment outcomes from the HCHF-BC (*n* = 52)

Item on HCHF-BC	Pre		Post		95% Confidence Interval of the Difference		*p*-value
	Mean	SD	Mean	SD	Lower	Upper	
Autonomy	3.25	1.55	3.71	1.35	−1.03	0.09	0.096
Family Meals	4.08	1.28	3.78	1.40	−0.05	0.64	0.096
Fruit Availability	4.30	0.91	4.58	0.70	−0.49	−0.07	0.009*
Energy Dense Snack Availability	2.39	1.27	1.80	0.87	0.27	0.92	0.001*
Fast Food Availability	1.65	0.77	1.33	0.55	0.12	0.51	0.003*

*p ≤ 0.01 denoted statistically significant difference between pre/post measures from the HCHF Behavior Checklist

Parents who completed the evaluation survey (*n* = 20) were very satisfied with the intervention. For example, they all selected ‘agree’ to the following statements; ‘I enjoyed coming to HCHF sessions’, ‘I would recommend HCHF to my friends and family’, ‘What I learned in HCHF is useful for me and my family’, and ‘I learned new parenting skills that help me get along better with my children.’ For questions pertaining to time and location, 95% of participants agreed that the time that the sessions were held was convenient for them, while 85% agreed that the location was convenient for them. Through open-ended questions, participants shared what they liked the most of the intervention which included being able to make changes in their homes, learning about the importance of eating healthy meals and how to share them with their children, in addition to their shared experiences with other parents.

## Discussion

This pilot study assessed preliminary changes in parents’ use of parent-reported FPPs and diet and activity behaviors of parents and children pre/post participation in the evidenced-based HCHF intervention. Recruitment and retention of this population was a challenge. For parents that did complete the intervention, the frequency of parent-reported encouragement of balance and variety increased. Although changes in other FPPs (both responsive and non-responsive) were not significant, trends toward improvement in most FPPs were observed. There were also improvements in reported dietary intake and physical activity measures (fruit, vegetable, and soda intake for parents, and parent-reported low-fat dairy intake and physical activity for their children). Parents also reported changes in measures related to the home food environment, including a significant increase in fruit availability and a significant decrease in energy dense snack availability and fast food availability for their children. Given the pilot nature of this work and the high rate of attrition, results should be interpreted with caution. Future studies should consider testing the effectiveness of the HCHF intervention using an experimental design and exploring FPPs as possible mediators of healthy eating and obesity.

The HCHF curriculum highlights healthy eating patterns and teaches parents ways to encourage healthy eating habits with their children. The HCHF curriculum may have had the greatest impact on encouragement of balance and variety due to the intervention content and the parent’s ability to implement this practice in the home. Previous studies focusing on FPPs have targeted mostly non-responsive practices; for example, one longitudinal study found that non-responsive FPPs at 6, 12, and 24 months post participation in a parent-centered childhood obesity treatment program decreased significantly at each time point [36]. The longitudinal study had a longer treatment intervention compared to the present study and also focused on non-responsive FPPs; it is possible that it is easier for parents to extinguish non-responsive FPPs versus learning about new and responsive practices [20]. Using more responsive practices however supports the development of healthy eating, favorable diet quality and weight outcomes over time [13, 18, 19, 21–24, 36, 37]. It is possible that there was not as much change as expected for several of the responsive FPPs given that they may be harder practices to operationalize in the home setting such as role modeling or involving children. The mean baseline scores for these other practices were also relatively high to begin with (mean = 3.7–4.5), creating ceiling effects for these measures. In addition, although the CFPQ is a validated tool, it has not been validated in this specific population (i.e. Hispanic, low-income), and future validation with these populations is needed [34].

The improvements seen in reported parent and child diet and activity behaviors are similar to previous studies utilizing the HCHF intervention, where significant improvements in parent-reported parent and child diet behaviors, including significant increases for fruit, vegetable, and low-fat dairy intake, and significant reductions in parent soda intake were found [28, 29]. These findings are not surprising in light of the topics thoroughly covered during the curriculum, including the importance of fruit and vegetable intake and drinking water or milk instead of sugar-sweetened beverages [26–29]. Although the targeted population was different from previously published studies, the results from this pilot study are consistent and support the possible efficacy of this intervention in improving health behavior outcomes for parents and children, particularly in low-income, Spanish-speaking populations.

Certain aspects of this study require additional comment. It is well known that participant recruitment and retention can be challenging in health-related studies and programs that aim to reach both parents and children [38–42]. In the present study, the attrition rate of parents was 40%. Furthermore, parents that completed the study were older than non-completers. Our experience is similar to that of other researchers where recruiting and retaining low-income populations in health-related studies remains difficult, especially those “hardest to reach” [41, 43]. Evidence suggests that the most common barriers to recruitment and retention include socioeconomic status, education level, study location (school vs. home vs. community) and program/intervention targets (i.e. parents or children only vs. parent and children), all of which may have affected retention in the current study [39, 42]. Common reported barriers of parent participation in this study, included transportation, parents’ work schedules and competing demands on family time [38]. Despite these barriers, the participants that were engaged continued to return to sessions and in fact requested additional sessions and education. To try and overcome the recruitment and retention challenges, barriers to study participation were reduced by working with a local community clinic, providing child care, healthy meals, and in some cases, transportation to the intervention. In order eliminate the health disparities experienced by low-income, ethnic minorities, continued efforts to reduce participation barriers in research studies are needed. Innovative approaches, including comprehensive policies and evidence-based strategies to improve recruitment and retention is warranted [43].

Given the funding mechanism and pilot nature of the study, an experimental design was not feasible. Findings need to be interpreted with caution given the lack of a control group and high attrition rates; significant results may be attributable to other factors and not necessarily the intervention itself. For example, participants may have been subjected to the observer-expectancy effect, as the study was described to participants as a health program for parents with the aim to improve the health of their families. In addition, behaviors were self-reported by parents, and therefore actual behaviors were not observed. Despite these limitations, the study utilized an evidence-based curriculum in a community-based setting, and was able to reach an at-risk population. By targeting a population served by a free clinic in a low-income area, the intervention was able to reach Hispanic and low-income parents of children who are disproportionately at risk for obesity [1–3, 31, 44]. Future studies should continue to explore cost-effective intervention strategies to engage low-income parents and assess long-term changes in behavior.

## Conclusions

This pilot study found that participation in the HCHF intervention by a primarily low-income and Hispanic population increased the reported frequency of encouragement of balance and variety, a responsive FPP, which is associated with favorable weight status and diet habits in children [6–8, 12, 13, 21, 24].

However, the study is a pilot and would benefit from further randomized studies to examine evidence of effectiveness of this intervention in similar populations. Although there are several obesity prevention studies, few have specifically targeted or measured FPPs, and few have taken a family-based approach [4, 9, 10, 14, 16]. Interventions to prevent childhood obesity may include some information on modifying FPPs, but few have had a comprehensive focus and/or have not measured changes in these practices pre/post intervention [36, 37, 45, 46].

Future interventions should focus on improving both responsive and non-responsive FPPs [6, 11–13, 19]. The results of the current study highlight the importance of possibly targeting those responsive FPPs and parenting behaviors surrounding the home food environment in health interventions aimed at reducing childhood obesity risk. These results add to the current literature on interventions focused on FPPs in a population at higher risk for obesity, by a targeting low-income, Hispanic population.

## Abbreviations

BMI: Body mass index
CEHC: Clinica Esperanza/Hope Clinic
CFPQ: Comprehensive feeding practices questionnaire
FPP: Food parenting practice
HCHF: Healthy children, healthy families
HCHF-BC: Healthy children, healthy Families behavior checklist
U.S.: United States
